# Structure, Function and Networks of Transcription Factors Involved in Abiotic Stress Responses

**DOI:** 10.3390/ijms14035842

**Published:** 2013-03-13

**Authors:** Søren Lindemose, Charlotte O’Shea, Michael Krogh Jensen, Karen Skriver

**Affiliations:** 1 Biomolecular Sciences, Department of Biology, University of Copenhagen, Ole Maaloes Vej 5, DK-2200 Copenhagen N, Denmark; E-Mails: slindemose@bio.ku.dk (S.L.); coshea@bio.ku.dk (C.O.); 2 Functional Genomics, Department of Biology, University of Copenhagen, Ole Maaloes Vej 5, DK-2200 Copenhagen N, Denmark; E-Mail: mikjensen@bio.ku.dk

**Keywords:** abiotic stress, transcription factor, gene regulatory network, interactome, protein intrinsic disorder, genetic engineering of crops

## Abstract

Transcription factors (TFs) are master regulators of abiotic stress responses in plants. This review focuses on TFs from seven major TF families, known to play functional roles in response to abiotic stresses, including drought, high salinity, high osmolarity, temperature extremes and the phytohormone ABA. Although ectopic expression of several TFs has improved abiotic stress tolerance in plants, fine-tuning of TF expression and protein levels remains a challenge to avoid crop yield loss. To further our understanding of TFs in abiotic stress responses, emerging gene regulatory networks based on TFs and their direct targets genes are presented. These revealed components shared between ABA-dependent and independent signaling as well as abiotic and biotic stress signaling. Protein structure analysis suggested that TFs hubs of large interactomes have extended regions with protein intrinsic disorder (ID), referring to their lack of fixed tertiary structures. ID is now an emerging topic in plant science. Furthermore, the importance of the ubiquitin-proteasome protein degradation systems and modification by sumoylation is also apparent from the interactomes. Therefore; TF interaction partners such as E3 ubiquitin ligases and TF regions with ID represent future targets for engineering improved abiotic stress tolerance in crops.

## 1. Introduction

Plants are constantly being exposed to abiotic stresses such as drought, high salinity, high osmolarity, threshold temperatures, nutrient deficiency, oxidation, and changing light conditions. These environmental stress factors negatively affect growth and productivity, and plants have evolved different mechanisms to respond to such challenges. At the molecular level this involves induction of stress-responsive and stress-tolerance genes [1], often mediated by the phytohormone abscisic acid (ABA). ABA is referred to as the plant stress hormone because, in addition to its role in development, it plays a key role in responses to abiotic stress factors by regulating stomatal closure to optimize transpiration, and by triggering the activation of many stress-related genes [2,3].

In plant genomes approximately 7% of the coding sequences are assigned to transcription factors (TFs) [4], and many of these are immediate-early abiotic stress-responsive genes [5]. These TFs probably initiate the indirect-late phase of responses by binding to *cis*-acting elements in the promoters of specific target genes encoding proteins with specific functions in for example protein turnover, the dehydration response and cell wall modifications [5]. Some of these TFs are master regulators of signaling and regulatory pathways of stress adaptation, and genetic engineering of one or a few of these may be sufficient to enhance stress tolerance in plants, making these TFs attractive targets for engineering [6].

**Figure 1:**
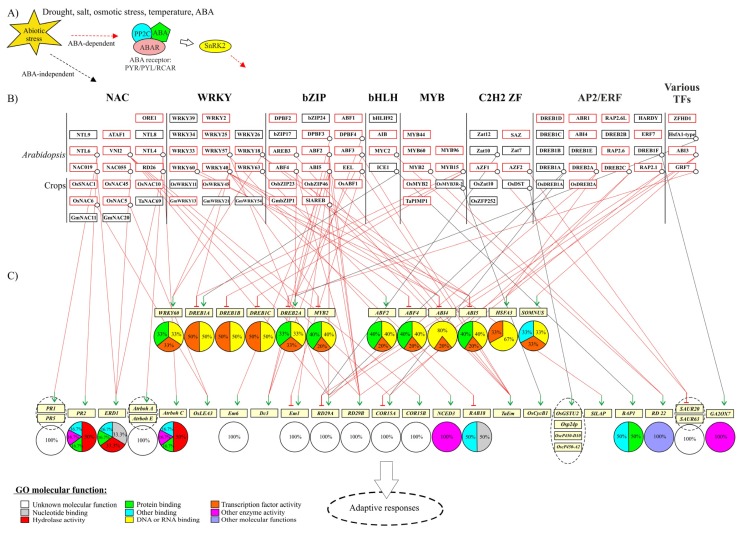
Gene regulatory networks of plant transcription factors (TFs) in plant abiotic stress responses and abscisic acid-dependent gene expression. (**A**) Drought, salt, osmotic stress, temperature, and ABA stress factors modulating the level and activity of the TF (**B**) and their target genes (**C**). ABA-dependent signaling pathway can involve TF phosphorylation through the PYR/PYL/RCAR ABA receptor [15]. (**B**) The boxes represent TF proteins from the model plant *Arabidopsis* or from different crop plants. Red boxes and lines show that the TF is associated with ABA-signaling. For TF and target genes with several names only one of the names is shown. For references and abbreviations used see the main text. (**C**) The direct target genes are divided into regulatory genes including TF genes and stress-responsive genes encoding functional proteins. The Gene Ontology (GO) molecular function term is shown as a circle with color code below each *Arabidopsis* target gene. The GO molecular function annotation was obtained using the Gene Ontology search tool [16] at The *Arabidopsis* Information Resource. Green arrows and red bars denote TF activation and repression, respectively. Some target genes with no known GO-based molecular function were grouped (*i.e.*, *PR1* and *PR5*, *Atrboh A* and *Atrboh E*, *SAUR20* and *SAUR63*, but also rice genes involved in ROS/H_2_O_2_ homeostasis, such as *OsGSTU2*).

Several recent reviews have addressed the role of specific TF families in abiotic stress responses [7–14]. In this review, the focus will be on selected TFs from several large plant TF families, which have been shown to play significant roles in responses to the important abiotic stress factors, including drought, high salinity, high osmolarity, threshold temperatures and the chemical abiotic stress factor ABA (Figure 1A). We will address the following questions: (1) What are the molecular mechanisms of principal TFs in abiotic stress responses? (2) What are their target genes and gene regulatory networks? and (3) How do they function in protein-protein interactions (interactomes), and what is the role of protein intrinsic disorder (ID) in these interactomes?

## 2. Large Plant TF Families in Abiotic Stress Responses

Several families of plant TFs play significant roles in translating abiotic stress signals into changes in gene expression. So far, research into TFs that regulate abiotic stress responses has mainly focused on single TFs and their isolated function. However, it is becoming increasingly clear that TFs also function as hubs, which have many partner proteins, in dynamic networks and as nodes between different pathways. This is also the picture emerging from analysis of TFs from the large TF families basic leucine zipper (bZIP), APETALA 2/ethylene-responsive element binding factor (AP2/ERF), NAM/ATAF1/CUC2 (NAC), WRKY, MYB, Cys2(C2)His2(H2)-type zinc fingers (ZFs), and basic helix-loop-helix (bHLH). A significant fraction of their members have been characterized with respect to their roles in the regulation of abiotic stress responses, and ectopic expression of several TFs from these families has resulted in improved crop stress tolerance. This section describes TFs which by forward and/or reverse genetic screens have been shown to play a functional role in plant stress responses and their gene regulatory networks, inferred from their direct promoter binding. Figure 1 provides an overview of the networks described in this section with relevant abiotic stress factors (Figure 1A), TFs known to function in abiotic stress responses (black boxes and lines) and/or ABA-associated signaling (red boxes and lines) (Figure 1B), and their direct target genes (Figure 1C).

### 2.1. bZIP TFs

The approximately 75 members of the *Arabidopsis* (*Arabidopsis thaliana*) bZIP TF family are divided into more than ten groups [17]. Many of the well-studied group A bZIP TFs play a central role in ABA signaling [10] (Figure 1B,C). For example, the ABA responsive element (ABRE) binding proteins/factors (AREBs/ABFs) *AREB1/ABF2*, *AREB2/ABF4*, *ABF1*, and *ABF3* are mainly expressed in vegetative tissues and all except *ABF1* are key regulators of ABA signaling that respond to osmotic stress during vegetative growth [10]. Overexpression of *AREB2/ABF4* or *ABF3* in *Arabidopsis* conferred ABA hypersensitivity, reduced transpiration, and enhanced drought tolerance [18], and overexpression of an activated form of *AREB1/ABF2* also showed increased ABA sensitivity and drought tolerance [19]. These three *AREB*/*ABF* TFs from both *Arabidopsis* and rice (*Oryza sativa*) require ABA for full target gene activation, and the *areb1 areb2 abf3* triple mutant displayed ABA hyposensitivity and reduced tolerance to drought stress compared to single and double *AREB*/*ABF* knockout mutants, suggestive of cooperative action between the three TFs [20,21]. The triple mutant also displayed differential expression of multiple stress-responsive genes, including late embryogenesis-abundant (LEA), group-A type-2C phosphatase (PP2C) and various TF genes [21]. AREB1/ABF2 and AREB2/ABF4 directly activated the expression of the ABRE-containing *R**esponsive to* *D**essication* (*RD29B*) promoter [20,21], and AREB1/ABF2, AREB2/ABF4*,* and ABF3 all bound to and activated the *DRE-BINDING PROTEIN 2A* (*DREB2A*) promoter in an ABRE-dependent manner. An ABA-dependent pathway was therefore suggested to play a role in the osmotic stress-responsive expression of *DREB2A*, which is mostly associated with ABA-independent stress-regulation [22].

The five *ABI5/DPBF* group A bZIP TFs, *ABI5*, *e**nhanced* *E**m* *l**evels* (*EEL*), *Dc3 promoter-binding factor 2 (DPBF2)/bZIP67*, *DPBF4*, and *AREB3*, are mainly expressed in seeds and control ABRE-mediated transcription [10]. ABA mediates phosphorylation and activation of these TFs to regulate the ABRE-dependent gene expression of importance to osmotic adjustment and seed dormancy, as well as seedling growth arrest [10,23]. *ABI5* was identified by forward genetic screens for mutants exhibiting ABA-resistant germination [24]. Plants overexpressing *ABI5* in a transient transactivation experiment resulted in strong and weak activation of the *RD29B* and RD29A promoters, respectively [25]. ABI5 and its close paralogues also bind and activate additional *LEA* class genes. Thus, ABI5, DPBF3, and DPBF4 bound to the *Arabidopsis Dc3* promoter [26], and ABI5, ABF3, AREB2/ABF4, AREB3, and EEL activated expression from the wheat (*Tritium aestivum*) *Em* promoter in transient overexpression assays, both in the absence and presence of ABA [27]. Interestingly, ABI5 and EEL showed antagonistic action by competing for the same binding site within the *Arabidopsis Em1* promoter [28], and chromatin immunoprecipitation (ChIP) experiments revealed that ABA increased ABI5 occupancy on the *Em6* promoter [29]. In conclusion, the *AREB/ABF* and *ABI5/DPBF* TF genes are key regulators of ABA-mediated ABRE-dependent gene expression.

Additional bZIP TFs are implicated in abiotic stress responses. For example, salt-stress induced proteolysis and translocation from the endoplasmatic reticulum to the nucleus of the group B TF bZIP17 has been observed. This is followed by upregulation of salt stress genes, which was blocked by a T-DNA insertion mutation in the *AtbZIP17* gene [30]. Furthermore, transcriptional repression of *AtbZIP24*, of Group F bZIP, using RNA interference, improved salt tolerance in *Arabidopsis*[31].

Relatively few bZIP regulators have been explored as potential candidates for application in the improvement of drought tolerance in crops. However, the Group A TF *Os*ABF1 from rice (*Oryza sativa*) and *Sl*AREB from tomato (*Solanum lycopersicum*) both enhanced tolerance to drought and salt stress [32,33], and *SlAREB* was found to bind to and activate transcription from the *Arabidopsis RD29B* and the tomato *leucine aminopeptidase* (*LAP*) promoters in an ABA-dependent manner [33].Furthermore, *OsbZIP23* overexpression conferred ABA hypersensitivity, and increased salinity and drought tolerance of rice [34], and overexpression of *OsbZIP46* increased ABA sensitivity. However, positive regulation of drought and osmotic stress tolerance by *Os*bZIP46 was dependent on its activation state [35].

The soybean (*Glycine max*) AREB/ABF *GmbZIP1* was highly induced by ABA, drought, high salt, and low temperature, and its overexpression enhanced the response of transgenic plants to ABA and triggered stomatal closure under stress conditions. Overexpression of *GmbZIP1* also enhanced the drought tolerance of transgenic wheat, suggesting that *GmbZIP1* may be useful for engineering stress tolerance for crops in general [36].

### 2.2. AP2/ERF TFs

The AP2/ERF plant-specific family with approximately 145 members in *Arabidopsis* contains four major subfamilies named AP2, RELATED TO ABI3/VP1 (RAV), ERF and dehydration-responsive element-binding protein (DREB). The DREB subfamily proteins play significant roles in abiotic stress responses by regulating gene expression via the *cis*-acting dehydration-responsive element/C-repeat (DRE/CRT) element [11] (Figure 1B,C). The DREB1 (A-1) sub-group consists of six members, mentioned below. *DREB1A/**C**-repeat-**b**inding* *f**actor 3 (CBF3)* was identified from its recognition of the DRE/CTR *cis*-acting element in the *RD29A/COR78/LT178*[37] and *COR15A* promoters [38]. *DREB1A/CBF3*, *DREB1B/CBF1*, and *DREB1C/CBF2* were all induced by cold, although the expression pattern of *DREB1C/CBF2* differed from that of the other two genes. Furthermore, DREB1B/CBF1 and DREB1A/CBF3 positively regulated the same set of target genes and were functionally distinct from DREB1C/CBF2 in cold acclimation [39]. *DREB1D/CBF4* is responsive to drought stress and, unlike other *DREB1/CBF* genes, also responsive to ABA [11,40]. *DREB1E/DDF2* and *DREB1F/DDF1* were both up-regulated by salinity stress, and *DREB1F/DDF1* directly upregulated the expression of the gibberellin-deactivating gene, *GA2ox7* under high-salinity stress in *Arabidopsis.* This resulted in reduction of the endogenous gibberellic acid (GA) level, repressed growth and improved stress adaptation [41].

The DREB2 (A-2) subgroup consists of eight members in *Arabidopsis* with DREB2A as a well-characterized transcriptional regulator. The *DREB2A* gene was only slightly up-regulated by ABA and both *DREB2A* and *DREB2B* were strongly induced by drought, salt, and temperature stress [37,42]. Transgenic plants overexpressing a constitutively active form of DREB2A (*DREB2A CA*), which lacks a negative regulatory domain (NRD), displayed improved tolerance to drought, high salinity, and heat shock, although the plants revealed growth retardation. Genes up-regulated by DREB2A were induced either by heat shock, drought or both [43,44]. In agreement with this, DREB2A bound both to the *RD29A*[37] and *h**eat* *s**hock* *f**actorA3* (*HsfA3*) promoters. DREB2A also activated the *HsfA3* promoter, and the expression of HsfA3 in turn induced the expression of *heat shock protein* (*Hsp*) genes [45]. Heat-induced *DREB2C* also bound and induced the *HsfA3* gene, indicating that DREB2A, DREB2C and HsfA3 cooperate in regulating heat tolerance in *Arabidopsis*[46]. The DREB2C overexpression lines also displayed altered stress response; while the plants were dehydration sensitive, they showed increased heat and freezing tolerance and were ABA-hypersensitive in accordance with the binding to and up-regulation of the promoters of both *HsfA3* and *COR15A*[47].

Other DREB subgroup genes have been reported to be stress-responsive and confer stress tolerance in transgenic plants. Thus, overexpression of *ABI4* of subgroup A-3 resulted in ABA-dependent expression of more than a hundred genes, with a synergistic effect between *ABI4* and several ABA-responsive bZIP TFs, including *ABI5*[48], and a subgroup A-5 member RELATED TO AP2.1 (RAP2.1) acted as a transcriptional repressor to keep cold and drought stress responses under tight control. In line with this, ChIP analysis identified the *Cold-regulated15A* (*COR15A*) and *RD29A* as *in vivo* downstream targets of RAP2.1 [49]. The ABA-responsive ERF subfamily member RAP2.6 and RAP2.6L play a dual role in abiotic and biotic stress responses, and overexpression of RAP2.6L enhanced performance under salt and drought stresses, without affecting the phenotype [50]. Another ERF subfamily member *AP2-like ABA repressor 1* (*ABR1*) was responsive to ABA and several abiotic stress conditions, and repressed ABA-regulated gene expression [51]. ERF7 also plays an important role in ABA responses, and is part of a transcriptional repressor complex [52].

Several studies have analyzed the DREB1 TFs for their ability to improve rice drought stress responses (for review see [53]). Although growth defects may result from overexpression of DREB1/CBFs using constitutive promoters [53], overexpression of *DREB1A/CBF3* using a stress-inducible promoter significantly improved spikelet fertility and increased the yield of transgenic rice compared to wild type plants under stress field conditions [54], and ectopic expression of *Arabidopsis* HARDY in rice improved water use efficiency without a reduction in growth [55]. Induced overexpression of *OsDREB2A* also proved successful with respect to improved survival rates of the transgenic rice plants under severe drought and salt conditions [56].

### 2.3. NAC TFs

The NAC family constitutes one of the largest plant-specific TF families with approximately 110 genes in *Arabidopsis*[57] and 150 genes in rice [58]. NAC TFs have a variety of important functions in plant development and abiotic stress responses [12,13,59] (Figure 1B,C), and whole-genome expression profiling studies in *Arabidopsis* have shown that most NAC genes are induced by at least one type of abiotic stress signal, such as salinity, drought, cold, or ABA [57].

Some of the well-characterized abiotic stress-associated NAC TFs belong to sub-group III-3, also referred to as ATAF [57] or SNAC (Stress-responsive NAC) sub-group [58]. Members of this sub-group, *ANAC019*, *ANAC055*, and *RD26 (ANAC072)*, were induced by drought, high salinity, ABA and JA [57,60], and shown to bind the promoter of *EARLY RESPONSIVE TO DEHYDRATION STRESS 1* (*ERD1*) [60]. Overexpression of these three NAC TFs resulted in up-regulation of several stress-inducible genes and improved drought tolerance of the transgenic plants [60]. Interestingly, up-regulation of the *ERD1* gene depended on co-overexpression of one of these NAC TFs and the ZF homeodomain transcriptional activator ZFHD1 [61] suggesting the existence of cooperative regulation of stress responses by members of different TF families. Ectopic expression of *ANAC019* and *RD26* resulted in ABA-hypersensitivity, suggesting that ANAC019 and RD26 are positive regulators of ABA signaling [57,60,62]. Overexpression of ATAF1 also affected plant tolerance to drought, although contradicting reports suggested positive and/or negative regulatory effects [63,64]. Several of the NAC TFs mentioned above are also implicated in other biological functions. For example, ANAC019 and ANAC055 also regulated jasmonic acid (JA)-induced expression of some defense genes. The *anac019 anac055* double mutant plants showed attenuated JA-induced expression of important wound responsive genes, and both JA- and ABA-induced expression of *ANAC019* and *NAC055* was dependent on the essential JA-signaling regulators, CORONATINE INSENSITIVE 1 (COI1) and MYC2. ANAC019 and ANAC055 may therefore be involved in crosstalk between the JA and ABA pathways for this induction [65,66]. Taken together, subgroup III-3 NAC TFs play important roles in abiotic stress perception and individual NAC members display functional overlap in stress responses.

NAC TFs from other NAC TF groups have also been implicated in abiotic stress responses. Namely, *ORE1/AtNAC2/ANAC092* of sub-group II-3 [57], which is involved in lateral root development, was also induced by salt stress and ABA [67], and plays a role in salt-promoted senescence [68]. In addition, the gene *VND INTERACTING2* (*VNI2*; sub-group III-1) was induced by high salinity in an ABA-dependent manner, and overexpression of *VNI2* enhanced resistance to salt stress and prolonged leaf longevity, as was also the case for overexpression of the direct target genes *COR15A*, *COR15B*, *RD29A*, and *RD29B*[69]. Thus, VNI2 integrates ABA-mediated abiotic stress signals into ageing. Furthermore, VNI2 is an interaction partner of the NAC TF VASCULAR-RELATED NAC-DOMAIN7 (VND7), which is a master regulator of xylem vessel differentiation and is related to programmed cell death [70], suggesting that stress tolerance, senescence and programmed cell death are connected via a NAC TF network containing VIN2 and VND7. Together, these examples illustrate how abiotic stress-responsive NAC TFs may be convergence points between functionally different pathways.

Fourteen transmembrane NAC TFs were predicted to be encoded in *Arabidopsis*[59], some of which are involved in stress responses. Recently the plasma membrane-anchored NTL6 of sub-group I was reported to be proteolytically processed upon exposure to cold. Activated NTL6 migrated to the nucleus and directly induced the *Pathogenesis-Related* (*PR*) genes, *PR1*, *PR2*, and *PR5* directly [71]. Interestingly, ABA simultaneously induced *NTL6* expression and NLT6 processing, and transgenic *Arabidopsis* plants overexpressing *NTL6* without the trans-membrane region (35S:6ΔC) were hypersensitive to ABA and high salinity during seed germination [72]. Closely related NAC TFs NTL8 and NTL9 regulated GA-mediated salt signaling in *Arabidopsis* during seed and leaf senescence, respectively, in response to osmotic stress [73,74]. Furthermore, the ABA, drought and heat inducible *NTL4* gene promoted reactive oxygen species (ROS) production during drought-induced senescence in *Arabidopsis*, and the expressed protein NTL4 directly bound and up-regulated the *Atrboh* (A, C, and E) genes encoding ROS biosynthetic proteins [75]. In conclusion, several membrane-anchored NTL NAC TFs are nodes connecting different signaling pathways of abiotic and biotic stress responses.

Several studies have addressed the role of crop NAC TFs in abiotic stress responses, and overexpression of SNAC/III-3 sub-group NAC TFs in rice has been especially successful with respect to enhancing stress tolerance. Overexpression of *SNAC1* enhanced drought resistance in transgenic rice in the field at the reproductive stage, and also improved drought resistance and salt tolerance in the vegetative stage. Compared to the wild type, the transgenic plants were more hypersensitive to ABA, and water loss was slower due to increased stomatal closure [76]. Overexpression of ABA-inducible *OsNAC6/SNAC2* and *OsNAC5* increased tolerance to dehydration and high salinity, although with growth retardation and low reproductive yields [77,78], and high salinity [79], respectively. Both NAC TFs bound to the promoter of the stress-inducible *OsLEA*3 gene [79]. Furthermore, overexpression of ABA-inducible *OsNAC10*, a subgroup III-2 NAC member, and *OsNAC45*, a subgroup II-3/CUC member, also enhanced abiotic stress tolerance of rice, in the case of *OsNAC10* also under field conditions [80,81]. Finally, overexpression of NAC genes also resulted in increased stress tolerance in other crops such as bread wheat (*Triticum aestivum; TaNAC69*) [82] and soybean (*Glycine max; GmNAC20* and ABA-inducible *GmNAC11*) [83].

### 2.4. WRKY TFs

The WRKY TF family, which also constitutes one of the largest plant TF families, is divided into three groups, based on the number of WRKY domains and the features of the associated zinc-finger-like motif [84]. WRKY proteins have been particularly associated with the regulation of plant pathogen responses. However, recent functional analyses have also implicated WRKY TFs in abiotic stress responses [7,14] (Figure 1B,C). For example, overexpression of *WRKY25* or *WRKY33* increased both salt tolerance and ABA sensitivity [85]. Modulation of the expression of *WRKY25, WRKY26*, and *WRKY33*, all group I *WRKY* genes, also affected resistance to heat stress through modulation of transcriptional reprogramming of heat-inducible genes, and the *wrky25*,*wrky26*, *wrky33* triple mutant was significantly more sensitive to heat stress than wild type plants [86]. WRKY25 and WRKY33 are also implicated in pathogen defense responses [87], putatively implicating these WRKY TFs in crosstalk between different signaling pathways. Furthermore, in relation to heat stress, heat stress-induced WRKY39, a group II WRKY protein, positively regulated the cooperation between the salicylic acid (SA) and JA activated signaling pathways that mediate responses to heat stress [88]. On the other hand, WRKY34 negatively mediated cold sensitivity of mature *Arabidopsis* pollen, possibly through the CBF cascade [89].

WRKY TFs also function as key components in ABA-mediated stress signaling. Recently, forward genetics analysis showed that WRKY57 improved drought tolerance in *Arabidopsis* by increasing the ABA level and up regulating stress-responsive genes. In accordance with this, ChIP assays showed that WRKY57 bound directly to the promoter of *RD29A* and the key ABA biosynthesis gene *9**-**c**is-**e**poxycarotenoid* *d**ioxygenase3* (*NCED3*) [90]. A T-DNA insertion mutant of the group III WRKY TF, *WRKY63/ABO3*, conferred ABA-hypersensitivity during seedling establishment and germination. Furthermore, the *abo3* mutation also impaired ABA-induced stomatal closure resulting in increased sensitivity to drought stress of the mutant plants compared to wild type plants [91]. WRKY63/ABO3 bound to the W-box in the promoter of *AREB1/ABF2* in accordance with repressed expression of the *AREB1/ABF2* gene in *abo3* mutant plants [91], and the ABA-dependent induction of WRKY63/ABO3 was impaired in the *abi1*, *abi2*, and *abi5* mutants. Together, these data showed that WRKY63 is on of the central components of the ABA-dependent gene regulatory network also involving group A ABRE-dependent bZIP TFs.

The closely related group II WRKY TFs, WRKY18, WRKY40 and WRKY60 all function as regulators of ABA signaling in seed germination and postgermination growth [92,93]. WRKY40 inhibited the expression of the important ABA-responsive genes *ABF4*, *ABI4*, *ABI5*, *DREB1A*, *MYB2*, and *RAB18* by directly binding to the W-box *cis*-acting element of these promoters [93]. Recently it was shown that not only WRKY40 but also WRKY18 and WRKY60 interacted with the promoters of *ABI4* and *ABI5* genes and cooperatively inhibited *ABI4* and *ABI5* expression. This regulation seems to be complex, and one WRKY TF may play either an agonistic or antagotistic role to other WRKY TFs in different situations [94]. This complexity is also clear from the study, in which disruption of *WRKY18* and *WRKY60* genes decreased ABA sensitivity, while disruption of *WRKY40* increased ABA sensitivity. The *WRKY18* and *WRKY60* mutants, but not the *WRKY40* mutant, were more tolerant to salt and osmotic stress than wild type plants. Adding to the complexity, both WRKY40 and WRKY18 bound to and activated the *WRKY60* gene, suggesting that *WRKY60* is a direct target gene of WRKY40 and WRKY18, in accordance with the induction kinetics of the three genes [92]. WRKY2 of group I also influenced seed germination and post germination growth, and *wrky2* knockout mutants displayed delayed or decreased expression of *ABI5* and *ABI3* and increased or prolonged expression of the *LEA* genes, *Em1* and *Em6*[95].

WRKY genes have also been explored for their ability to improve abiotic stress tolerance in crops. In rice, expression of *OsWRKY11* by the heat-inducible *HSP101* promoter resulted in enhanced drought and heat tolerance [96]. Interestingly, OsWRKY45 alleles play different roles in abscisic acid signalling and salt stress tolerance, but similar roles in drought and cold tolerance in rice [97]. Overexpression of *GmWRKY21* and *GmWRKY54* in *Arabidopsis* resulted in increased tolerance to cold stress and salt and drought stresses, respectively, whereas overexpression of *GmWRKY13* resulted in increased sensitivity to salt and mannitol, but decreased sensitivity to ABA [98], making these genes candidates for improving stress responses, also in soybean (*Glycine max*).

### 2.5. Cys2His2 Zinc Finger (C2H2 ZF) TFs

The *Arabidopsis* genome encodes about 176 Cys2(C2)His2(H2)-type ZFs [99], and C2H2 ZF proteins with an ERF-associated amphiphilic repression (EAR) domain are important transcriptional repressors regulating responses to environmental stress factors [8,100]. Many of these belong to subclass C1-2i, and the members contain two dispersed C2H2-type Zn fingers [8,99]. Several Zat proteins are implicated in abiotic stress responses (Figure 1B,C). Thus Zat10/STZ, the gene of which is cold induced, bound to the promoter of and repressed the *RD29A* gene [101], and constitutive expression of *Zat10* in *Arabidopsis* enhanced the tolerance of plants to salinity, heat and osmotic stress. Surprisingly, knockout and RNAi mutants of *Zat10* turned out to be even more tolerant to osmotic and salinity stress [102]. By contrast, overexpression of *Zat7* also resulted in increased tolerance to salinity stress [100]. On the other hand, overexpression of *Zat12* in *Arabidopsis* caused a minor increase in freezing tolerance, and Zat12 downregulated the expression of the *DREB/CBF* genes suggesting that Zat12 is a negative regulator of the CBF cold response pathway [103].

*AZF1* and *AZF2*, also subclass C1-2i members, were induced by osmotic stress and ABA, and overexpression of these genes compromised plant growth and viability. AZF1 and AZF2 repressed a set of genes that were downregulated by osmotic stress and ABA treatment and many auxin (IAA)-responsive genes, and AZF1 and AZF2 bound the promoter of two of these, SAUR63 and SAUR20, in electrophoretic mobility shift assays (EMSAs). This suggested that AZF1 and AZF2 function as transcriptional repressors to inhibit plant growth, for example by inhibiting auxin-mediated plant growth under abiotic stress conditions [104]. Another EAR-motif C2H2 ZF, *S**A- and* *A**BA-downregulated* *z**inc finger* gene (*SAZ*) also negatively regulated a subset of ABA-responsive genes, including *RD29B* and *RAB18* in *Arabidopsis* under unstressed conditions [105].

Similar to the function of *Arabidopsis Zat10*, the overexpression of *OsZat10*, which itself is induced by *Os*DREB1A/CBF3, conferred drought tolerance and, importantly, a 17%–36% yield increase under rain-free conditions [54]. Recently the C2H2 ZF TF *DROUGHT AND SALT TOLERANCE* (*DST*) was shown to control stomatal aperture under drought and salt stress in rice by direct modulation of genes related to H_2_O_2_ homeostasis. In accordance with this, DST was associated with the promoter sequence of the *peroxidase 24 precursor (Osp24p) Osglutathione-S-transferase2 (OsGSTU2)*, *cytochrome P450 71D10* (*OscP450-D10*), and *cytochrome 9450 94A2* (*OscP450*) genes in ChIP assays [106]. Overexpression of a type TFIIIA C2H2 ZF, *ZFP252*, enhanced the tolerance of rice seedlings to drought, and this tolerance was correlated with the induction of *OsDREB1A* and with a higher accumulation of free proline and soluble sugars. Therefore, ZFP252 represents another candidate for engineering crop plants with enhanced tolerance to salt and drought stresses [107]. In a recent addition to this research field, the EAR-motif containing C2H2 ZF ZFP182 from rice was shown to mediate salt, cold, and drought tolerance [108].

### 2.6. MYB TFs

The *Arabidopsis* genome encodes 126 MYB TFs, characterized by sequence repeats R1, R2, and R3 of the MYB domain. The R2R3-MYB sub-family is highly expanded in plants [9], and several R2R3-MYB genes are implicated in ABA-mediated abiotic stress responses (Figure 1B,C). For example, *MYB60* is downregulated by ABA and dehydration stress and involved in regulation of stomatal opening [109], and *MYB44/MYBR1* also regulated ABA-mediated stomatal closure in response to abiotic stress. Furthermore, overexpression of *MYB44/MYBR1* resulted in suppression of JA-responsive gene activation. The hypothesis of mutually antagonistic actions between JA- and ABA-signaling pathways was therefore proposed [110,111]. *MYB96*, induced by drought and ABA, regulated drought stress responses by integrating ABA and auxin signals [112]. *MYB2* was inducible by ABA and dehydration, and transgenic *Arabidopsis* plants overexpressing both *MYB2* and the *bHLH* TF gene *MYC2/RD22BP1* exhibited hypersensitivity to ABA and enhanced osmotic stress response compared to wildtype plants [113]. Both proteins bound to and activated transcription from the dehydration-responsive gene *RD22*[114].

The *MYB15* gene is upregulated by cold stress, and MYB15 bound to Myb recognition sites in the promoter of *DREB1B/CBF1*, *DREB1C/CBF2*, and *DREB1A/CBF3* and downregulated these genes. In accordance with this, MYB15 reduced freezing tolerance [115]. MYB15 was also associated with additional abiotic stress responses, since overexpression of *MYB15* improved drought and salt tolerance in *Arabidopsis* and resulted in ABA-hypersensitivity [116].

Only a limited number of studies have analyzed the effect of ectopic *MYB* expression in crop plants. In a recent study, *OsMYB2* overexpressing plants were shown to be more tolerant to salt, cold, and dehydration stresses and more sensitive to ABA than wildtype plants [117]. Enhanced tolerance to cold stress in *OsMYB3R-2* transgenic rice was suggested to be mediated by alteration in cell cycle and ectopic expression of stress genes. Furthermore, *Os*MYB3R-2 bound directly to the promoter of the cycling gene, *OsCycB1*[118]. Recently the wheat MYB TF *Ta*PIMP1 was shown to be a positive molecular linker mediating resistance to both *Bipolaris sorokiniana* and drought stress by regulating stress-related genes in ABA- and SA-signaling pathways in wheat [119]. PIMP1 thereby represents one of several MYBs implicated in both biotic and abiotic stresses [9].

### 2.7. bHLH TFs

Proteins of the bHLH family are encoded by 162 genes in *Arabidopsis*[120], and a few of these play a role in ABA-signaling and abiotic stress responses (Figure 1B,C). ABA-inducible *MYC2/RD22BP1* was initially characterized as a positive regulator of ABA-inducible genes under osmotic stress conditions, and MYC2 functioned cooperatively with MYB2 TFs in transactivation of the *RD22* gene [113,114]. MYC2 has been referred to as the master of action because of its role in the regulation of crosstalk between the signaling pathways of JA and ABA, SA, GAs, and auxin (IAA) [121]. The ABA-inducible TF AIB positively regulated ABA responses in *Arabidopsis* and plants overexpressing *AIB* showed increased drought tolerance [122]. The *bHLH92* gene was induced by NaCl, dehydration, mannitol, and cold treatments, and overexpression of *bHLH92* resulted in a modest increase in tolerance towards NaCl and osmotic stresses through regulation of salt- and drought-responsive genes [123].

The well-characterized bHLH TF inducer of CBF expression 1 (ICE1) is a regulator of CBF genes during cold periods. The *ice1* mutation abrogated the expression of *DREB1A/CBF3* and decreased the expression of many genes downstream of CBFs, which resulted in a significant reduction in plant tolerance to cold and freezing [124]. ICE1 interacted with MYB15, and together these TFs bound to the *DREB1A*/*CBF3* promoter to regulate cold stress tolerance [115]. ICE1 also bound to the promoter of the *B**ON1-**a**ssociaited* *p**rotein**1* (*BAP1*) that is responsive to a moderate decrease in temperature, and is required for the cooling induction of *BAP1*. The *ice1* mutant showed a low level of induction of *BAP1* and enhanced resistance to a bacterial pathogen, possibly through potentiating SA-signaling [125].

### 2.8. Various TFs in Abiotic Stress Responses

Recently, an additional TF was reported to be involved in the DREB2A abiotic stress network (Figure 1B,C). A yeast one-hybrid screening for TFs binding to the *DREB2A* promoter identified GROWTH-REGULATING FACTOR7 (GRF7) as a repressor of *DREB2A*. GRF7 was suggested to function as a repressor of a broad range of osmotic stress and/or ABA-responsive genes to prevent growth inhibition normally associated with increased stress tolerance by DREB2A [126]. In relation to the involvement of DREB2A in heat stress, HsfA1-type TFs activated the transcription of the *DREB2A* gene, and the heat shock-responsive expression of *DREB2A* disappeared in *hsfa1 a/b/d* triple and *hsfa 1 a/b/d/e* quadruple mutants. The triple mutant showed greatly reduced tolerance to heat shock stress compared to wildtype plants [127]. The B3 domain TF ABI3, another well characterized TF, acted upstream of ABI5 to regulate ABA-dependent gene regulation during germination [29]. ABI3 regulated the *RD29B* and *RD29A* promoters strongly and weakly, respectively [25], and recent evidence showed that ABI3 also activated the promoter of SOMNUS, a key negative regulator of seed germination [128].

### 2.9. Gene Regulatory Networks in Abiotic Stress Responses

Figure 1 provides an overview of TFs, representing several large plant TF families, which play a functional role in responses to the important abiotic stress factors drought, high salinity, high osmolarity, temperature extremes and the stress hormone ABA. Most of these TFs are implicated in ABA-dependent signaling as shown by the red-boxed protein names in Figure 1B. ABA signaling is positively regulated by the PYRABACTIN RESISTANCE (PYR)/PYR1-LIKE (PYL)/REGULATORY COMPONENT OF ABA RECEPTOR (RCAR) ABA receptor (ABAR) (Figure 1A), which perceives ABA intracellularily and forms stable ternary complexes with clade A protein phosphatases 2C (PP2Cs). This inactivates the phosphatases, which allows autophosphorylation and activation of downstream subclass III sucrose non-fermenting 1 (SNF1)-related protein kinase 2 (SnRK2). The activated kinase phosphorylates and thereby activates the key components of ABA-signaling, the AREB/ABF TFs and ABI5 [15], which directly induce the expression of genes encoding, for example, LEA proteins and RD29A/B (Figure 1), contributing to stress tolerance and adaptation.

Figure 1C shows direct target genes, identified in ChIP experiments, EMSAs and transactivation assays, for the TFs shown in B. This can be used for assembly of putative gene regulatory networks. Interestingly, some of the AREB/ABF and ABI5-like TFs are themselves direct downstream targets of other TFs. Thus WRKY63 directly regulated *AREB1/ABF2*[91], whereas WRKY40 directly regulated *AREB2/ABF4* and *ABI5. ABI5* was also shown to be a direct downstream target of the close relatives of WRKY40, WRKY18 and WRKY60 [93,94] (Figure 1B,C). This suggests that some of the WRKY TFs are early nodes in ABA-signaling [14], with the LEA and RD29A/B proteins as putative functional proteins. Only genes which have so far been shown to be direct targets of the TFs in B are shown in Figure 1C. Due to this high level of stringency, components such as WRKY63 [14] may be missing from the emerging networks. Another interesting putative network is related to cold responses. Thus the functional genes *COR15A* and *RD29A* were identified as direct targets of DREB1A/CBF3 [37,38]. *DREB1A/CBF3*, on the other hand, is immediately downstream of the interacting cold-regulating TFs ICE1 and MYB15 [115]. MYB15 also directly targets additional DREB/CBF TFs and BAP1 [125]. Thus, this putative network involves cold-associated TFs from at least three major plant TF families. The figure also illustrates suggested crosstalk between abiotic stress signaling involving ABA-regulated NTL6, and biotic stress signaling involving the PR genes.

Some of the TF target genes shown in Figure 1C are direct targets of multiple TFs representing several TF families. For example, the well-studied *RD29A* and *RD29B* genes are common targets of both NAC, WRKY, bZIP, C2H2 ZF, AP2/ERF, and B3 domain TFs, suggestive of overlapping regulatory mechanisms and crosstalk between pathways involving, for example, drought, low temperature, osmotic stress, dehydration, and cold. Regulation of the *RD29A* and *RD29B* genes can also be both ABA-dependent and ABA-independent, which in the case of *RD29A* can be integrated through the DRE/CRT and ABRE *cis*-acting elements [129].

Figure 1C shows the Gene Ontology (GO) molecular function term for the TF target genes. A significant fraction of these are themselves TFs. However, many of the target genes also encode cell protection proteins such as the LEA proteins (LEA3, Em6, Dc3, Em1, COR15A, COR15B, RAB18, Em), which can function as chaperones [130], additional chaperones (*Sl*LAP), biosynthetic enzymes (NCED3), and cell cycle regulators (CycB1). In addition, although the TFs presented have been studied in relation to their function in abiotic stress responses several direct target genes (*PR1*, *2*, and *5*, *SAUR20* and *63*, and *GA2OX7*) have mainly been associated with different biological functions.

## 3. Structures of Abiotic Stress-Related TFs: Determinants of Function

**Figure 2:**
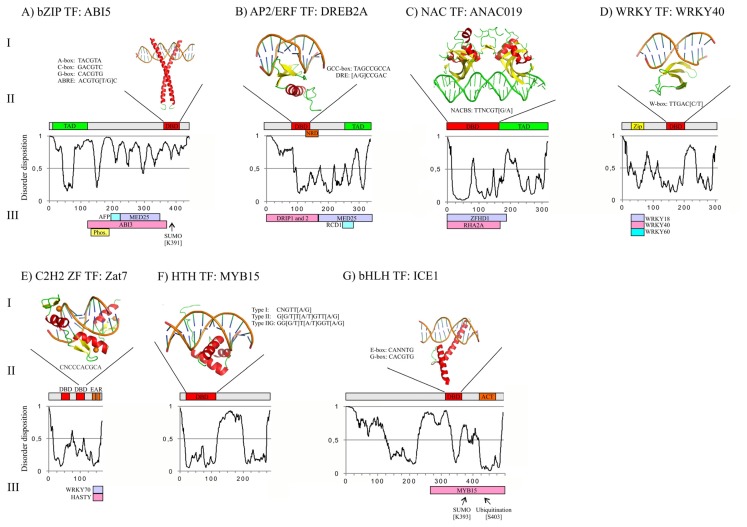
Structure of well-studied members of the bZIP, AP2/ERF, NAC, WRKY, C2H2 ZF, HTH and bHLH TF families. **(I**) Tertiary structure of the TF family designating DBD in complex with DNA. (**II**) Schematic primary structure drawn to scale and disorder predictions for a selected TF. (**III**) Regions involved in protein-protein interactions and post-translational modifications. (**A**) X-ray structure of the bZIP DBD from mouse CREB (PDB accession code 1DH3) and schematic representation of the bZIP TF ABI5. (**B**) NMR structure of the ERF DBD from *Arabidopsis* ERF1 (1GCC) and schematic representation of the AP2/ERF TF DREB2A. (**C**) X-ray structure of the NAC DBD from *Arabidopsis* ANAC019 (3SWP) and schematic structure of ANAC019. (**D**) NMR structure of the WRKY DBD from *Arabidopsis* WRKY4 (2LEX) and schematic representation of WRKY40. (**E**) X-ray structure of the C2H2 ZF DBD from mouse Zif268 and schematic representation of Zat7. (**F**) NMR structure of the HTH domain, part of the MYB DBD, from human TRF2 and schematic representation of MYB15. (**G**) X-ray structure of the bHLH DBD from mouse MyoD and schematic representation of ICE1.

The TFs discussed above are gene-specific DNA-binding regulatory proteins, which either activate or repress transcription of target genes. TFs are grouped into families based on the DNA-binding domain (DBD). TFs predominantly bind DNA in a sequence-specific manner via the DBD, thereby only targeting promoters with a given consensus sequence [131]. Apart from the DBD, TFs contain a transcription regulatory domain (TRD), most commonly a transcriptional activation domain (TAD), which has been classified on the basis of amino acid profile, e.g., as acidic, glutamine-, proline- or serine/threonine-rich. TRDs often have a high degree of low-complexity sequences and a propensity for flexible protein segments that fail to self-fold into an ordered three-dimensional structure, commonly referred to as intrinsic disorder (ID) [132]. Plant TFs of different families, e.g., NAC, bHLH, MYB, bZIP, WRKY, ZF, and AP2/ERF also have significant degrees of ID, which can play a functional role in interactions with other regulatory proteins, as exemplified by the interaction between the *C*-terminal domain of *Hv*NAC013 and Radical Induced Cell Death1 (RCD1) [133,134]. In this section, we describe structure-function aspects of significant members of the TFs families presented above. Figure 2 summarizes the information and for each TF known plant DNA-binding sites are integrated below the structures. An exception is mouse Zif268 where the DNA sequence shown is adapted from the UniProbe database (http://thebrain.bwh.harvard.edu/uniprobe/). Information about TAD, NRD, leucine zipper (zip), and bacterial aspartate kinase, chorismate mutase and TyrA (ACT) domain were identified by BLAST searches and literature mining. For POND-FIT [135] analysis a threshold is applied with disorder assigned to values ≥0.5.

### 3.1. bZIP DBD and Overall Structure of ABI5

The *Arabidopsis* bZIP TFs are divided into 10 groups based on sequence similarities of their basic regions. Members within the same group have additional features in common, such as the size of the leucine zipper and conserved sequence motifs [17]. Figure 2A shows the schematic structure of the extensively studied Group A bZIP TF ABI5. The bZIP DBD consists of two α-helices with a basic region that binds DNA, and a leucine zipper dimerization motif, as illustrated in Figure 2 by the X-ray structure of the bZIP from mammalian cAMP response element-binding protein (CREB) in complex with target DNA [136]. Plant bZIP proteins preferentially bind to DNA with an ACGT core with binding specificity being regulated by flaking nucleotides from the TACGTA (A-box), GACGTC (C-box) and CACGTG (G-box) [17] or ABRE (ACGTGT/GC) [137]. ABI5 is predicted to be remarkably disordered with the TAD (amino acids residues 9-122; [138]) mapping to regions with a large degree of the ID (Figure 2A). The RCD1-interacting bZIP TGA2 also contains large regions with ID propensity [134].

### 3.2. AP2/ERF DBD and Overall Structure of DREB2A

DREB2A is a well-studied member of the AP2/ERF TF family, and aspects of both the AP2/ERF DBD and the overall DREB2A structure have been reviewed recently (Figure 2B) [134,139]. AP2/ERF family members share a conserved AP2/ERF DBD of approximately 60 amino acid residues referred to as the GCC-box binding domain (GBD). The NMR solution structure of the AP2/ERF DBD of ERF1 in complex with GCC-box DNA (5′-TAGCCGCCA-3′) revealed that the AP2/ERF DBD contains an *N*-terminal, three-strand anti-parallel β-sheet that recognizes a target sequence and a *C*-terminal α-helix, which is packed parallel to the second beta-strand [140] (Figure 2B). The DNA sequences recognized and bound by the DBDs differ between the AP2/ERF subfamilies, and the DREB/CBF TFs also depend on short signature sequences both *N*-terminally and C-terminally of the DBD for binding to the DRE [141]. Members of the DREB and ERF subfamilies recognize similar, but slightly different sequences. For example, DREB/CBF TFs recognize the DRE sequence, 5′-[A/G]CCGAC-3′, and ERF TFs recognize the GCC-box sequence, 5′-AGCCGCC-3′ and variations thereof, respectively [11]. Members of the AP2 subfamily recognize longer sequences with a 5′-GCAC[A/G]N[A/T]TCCC[A/G]ANG[C/T]-3′ consensus [142], and the AP2/ERF domain of RAV1 recognizes the 5′-CAACA-3′ motif [143]. The recently published ID-prediction profile showed that DREB2A has a high degree of ID in both the *N*- and *C*-termini [134]. The transcription activation domain (TAD) of DREB2A maps to region 254 to 335 [43], which is part of a larger DREB2A fragment that was shown to lack secondary structure [144], and the NRD of DREB2A maps to region 136–165, [43], which on the other hand is predicted to be structured (Figure 2B).

### 3.3. NAC DBD and Overall Structure of ANAC019

The NAC DBD of ANAC019, shown by schematic structure in Figure 2C), was for a long time the only NAC domain for which the tertiary structure was known [145]. The X-ray crystal structure showed that the 168 amino acid residues NAC DBD forms a novel fold consisting of a twisted antiparallel β-sheet that packs against an *N*-terminal α-helix on one side and a short α-helix on the other side. This overall fold was also found by the recent determination of the rice SNAC1 NAC domain structure [146]. The strict consensus binding site for ANAC019, the NAC-binding site (NACBS), was identified using a reiterative selection procedure and shown to be 5′-TTNCGT[A/G]-3′ [147], in accordance with the core consensus CGT[G/A] identified for several other NAC proteins [13,60]. Although NAC TFs may target single NACBSs *in vivo*[60], palindromic NACBSs bind with higher affinity [147], and were therefore used for structure determination. Information from a combination of low resolution X-ray crystallography and biochemical solution studies showed that a β-strand with the highly conserved sequence WKATQTD sequence protrudes into the major groove of the DNA (Figure 2C) [148]. Interestingly, both GLIAL CELL MISSING (GCM) TFs and WRKY TFs also use a central β-sheet with similar topology for DNA binding. When the sheets were superimposed, the DNA-binding strand of NAC aligned with the DNA-binding strands of GCM and WRKY [139,149,150], providing solid support for a relationship between the NAC, GCM and WRKY TFs. The TAD of the NAC TFs map to the C-terminal part of the proteins, which were predicted to be disordered especially compared to the N-terminal NAC domain [57]. This is also the case for ANAC019 (Figure 2C), and ID of the TAD was experimentally verified for senescence-associated *Hv*NAC013 [133].

### 3.4. WRKY DBD and Overall Structure of WRKY40

Figure 2D shows the schematic structure of abiotic stress associated WRKY40, and the tertiary structure of the WRKY DBD of WRKY4. So far, the tertiary structure has not been determined for a WRKY domain from an abiotic stress-related WRKY TF. However, the structure of the 60 amino acid residues WRKY domain of WRKY1 and WRKY4 and the NMR solution structure of WRKY4 in complex with target DNA have been determined [139,151,152]. Figure 2D shows the position of the WRKY domain in the schematic structure of WRKY40. The WRKY domain consists of a four- or five stranded antiparallel β-sheet structure with a Zn-binding pocket formed by either C_X4–5_C_X22–23_H_X_H or C_X7_C_X23_H_X_C at the *N*-terminus [153]. A β-strand with the conserved WRKYGQK sequence enters the major groove of the target DNA and is involved in sequence-specific DNA binding [150]. The conservation of the WRKY domain is in accordance with a high degree of conservation of its cognate binding site the W-box 5′-TTGAC[C/T]-3′ [153], and mutations in both the WRKYGQK sequence and of the Zn-binding residues impaired DNA-binding [154]. In general WRKY TFs can activate or repress transcription, and they are rich in potential transcriptional activation and repression domains [84]. The schematic structure of WRKY40 suggests that this TF has several regions with ID, which represent putative TRDs and PPIs regions.

### 3.5. C2H2 ZF DBD and Overall Structure of Zat7

The 168 amino acid residues C2H2-type ZF TF Zat7 contain two C2H2 ZFs both with a conserved plant-specific DNA-binding QALGGH sequence (Figure 2E) [8]. The C2H2-motif consists of approximately 30 amino acid residues and includes two conserved Cys and His bound to one zinc ion. Each finger forms two β strands and one α helix, as shown by the X-ray structure of three ZFs from mouse Immediate-Early protein Zif268 [155]. As is typical for plant C2H2-type ZFs [8], the two fingers in Zat7 are separated by a long spacer predicted to be flexible (Figure 2E). *In vitro* binding analysis revealed that the conserved plant-specific QALGGH motif plays a critical role in DNA binding activity [156]. Surprisingly, the small Zat7 protein, which also contains two EAR repression domains [100], was predicted to be mostly structured (Figure 2E). This is in contrast to the large C2H2 ZF TF INDETERMINATE DOMAIN5 (IDD5), which was predicted to be structured only in the ZF region [134].

### 3.6. Helix-Turn-Helix (HTH) DBD and Overall Structure of MYB15

MYB15 is shown as a representative of the R2R3 MYB TFs which are highly proliferated in plants (Figure 2F) [9]. The MYB DBD is highly conserved and consists of sequence repeats (R) of about 52 amino acid residues, each forming three α-helixes. The second and third helices of each repeat form a helix-turn-helix (HTH) structure with a hydrophobic core in the HTH fold [157]. MYB TFs have binding specificity to either type I (5′-CNGTT[A/G]-3′) or type II (5′-G[G/T]T[A/T]GTT[(A/G]-3) and type IIG (5′-G[G/T]T[(A/T]GGT[A/G]-3′) MYB recognition sequences. MYB15 preferentially bound to type II and type IIG [115,158]. MYB15 contains one R2 and one R3 type sequence repeat in the MYB DBD. Since no R2R3 MYB-DNA complex structure has been determined, the HTH domain of the teleomeric DNA-binding protein TRF2 in complex with target DNA [159] is shown together with the schematic structure of MYB15 (Figure 2F). MYB15 is predicted to have a large disordered region in the middle of its sequence.

### 3.7. bHLH DBD and Overall Structure of ICE1

The bHLH TF ICE1 consists of 494 amino acid residues, and in addition the DBD it contains one recognizable domain, the bacterial aspartate kinase, chorismate mutase and TyrA (ACT) amino acid-binding (Figure 2G). The bHLH DBD consists of approximately 60 amino acids with two functionally distinct regions. The basic N-terminal region of the DBD is approximately 15 amino acid residues long and is involved in DNA-binding, whereas the C-terminal end functions as a dimerization domain and consists mainly of hydrophobic amino acid residues that form two amphiphatic α-helices separated by a loop region [160]. Co-crystal structural analysis showed that the interaction between the bHLH regions of two separate polypeptides leads to formation of homodimers and/or heterodimers and that the basic region of each partner binds to half of the DNA recognition site, as shown for the X-ray structure of the mouse MyoD bHLH domain bound to DNA [161] (Figure 2G). The core DNA sequence recognized by the bHLH proteins is a consensus hexanucleotide sequence known as the E-box (5′-CANNTG-3′). There are different types of E-boxes, and one of the most common ones is the palindromic G-box (5′-CACGTG) [160]. ICE1 bound to five different E-box variants in the *CBF3* promoter [124] and to the 5′-CAAATG-3′ sequence in the *BAP1* promoter [125]. ICE1 is predicted to have a significant degree of ID, as was also the case for the bHLH proteins PIF3 and bHLH011 [134].

### 3.8. Structure of TFs in Abiotic Stress Responses

Gene-specific TFs recognize DNA by their DBD, which is generally known to determine DNA-binding specificity. The DBDs of the TFs shown in Figure 2 all have well-defined tertiary folds, providing a scaffold. As expected, the DBD generally maps to regions predicted to be structured (Figure 2). However, many of the DBDs are surrounded by regions with high propensities for ID, and such flanking disordered regions have recently been suggested to affect DNA-binding specificity and affinity [162,163]. Interestingly, remote ID regions may fine-tune selectivity as well as affinity of DNA-binding through fuzzy (involving ID) interactions with the DNA binding surface [164]. All the TFs shown in Figure 2, except Zat7, were predicted to have a significant degree of ID, and ABI5 and ICE1 were predicted to be mostly disordered. Whether or how the disordered regions affect DNA-binding specificity remains to be determined. Regions with ID are extremely flexible, which forms the basis for their function. Many ID regions (IDRs) are multi-specific and can adapt to several diverse interactions partners. The large interaction potential allows IDRs to interact as hubs with many protein partners [165] in interactomes. The importance of ID to protein interactions is just becoming appreciated in plant science [134].

## 4. Regulation of TF Activity through Protein-Protein Interactions (PPIs)

Several of the TFs presented are key regulators of abiotic plant stress responses, and some of these have also proved successful in controlling stress responses in field-grown crops [53,54,76]. Even though many of the TFs function by enhancing stress adaptation, abiotic stress responses often interfere with normal growth and development. This makes proper control of the level and the activity of these TFs essential, and several mechanisms are used for this purpose. As shown by the many examples presented here, transcriptional regulation of gene expression by specific TFs binding to specific *cis*-acting elements in gene promoters represents a major level of control. It is also becoming increasingly clear that the efficiency of gene expression is influenced by the chromatin structure, which is regulated by processes such as DNA methylation and posttranslational modification of histones. The SWI2/SNF2 chromatin remodeling ATPase BRAHMA (BRM), which represses ABA signaling in the absence of stress stimuli in *Arabidopsis*, represents a recent example of this type of control. In accordance with this, *ABI5* expression was derepressed in drought tolerant *brm* mutants [166]. At the post-transcriptional level several of the TFs are potential regulatory targets of silencing by small RNAs (miRNAs). For example, miRNA164 has affinity for several NAC mRNAs [13], and four NAC targets of miRNA164 were identified as drought responsive in different *Populus* species [167]. Alternative splicing also represents a method of control, as shown for DREB2A [168]. Here we focus on post-translational control involving PPIs through analysis of the interactomes of the TFs selected and presented in section 3.

### 4.1. ABI5 Interactome and Regulation

**Figure 3:**
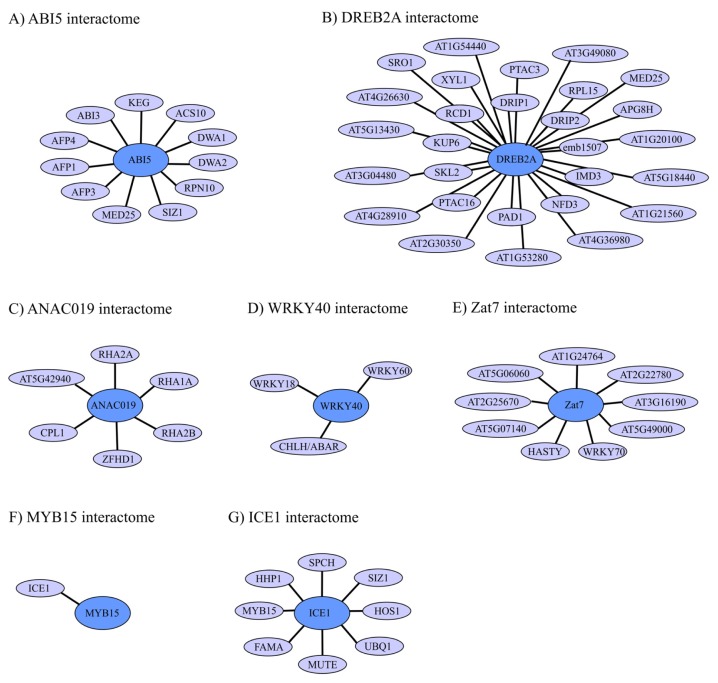
*Arabidopsis* interaction networks with different abiotic stress-related TFs as hub. The interactomes are visualized from the N-Browse viewer (http://www.arabidopsis.org/tools/nbrowse.jsp) [169] with manual modifications as indicated below (**A**) ABI5 interactome; (**B**) DREB2A interactome with addition of SRO1; (**C**) ANAC019 interactome; (**D**) WRKY40 interactome with addition of CHLH/ABAR; (**E**) Zat7 interactome; (**F**) MYB15 interactome; (**G**) ICE1 interactome.

ABI5 has a significantly sized interactome (Figure 3A) reflecting both positive and negative regulation of this key regulator of ABA-dependent stress responses. ABI5 is positively regulated through the PYR/PYL/RCAR ABA receptor (Figure 1) [15]. Thus, phosphorylation of ABI5 by SnRK2 (not shown in Figure 3A) in a region from amino acid residue 132 to 190 (Figure 2A) is a requirement for effective function as an ABA-dependent TF regulating downstream target genes [170,171]. Several negative regulators of ABI5 have been reported. The ABI five binding proteins (AFPs), identified as ABI5 interaction partners in yeast two-hybrid screenings, negatively affected ABA and salt sensitivity and the level of ABI5, possibly through ubiquitin-dependent proteasomal degradation [172,173], which plays a significant role in regulating the ABI5 level. ABI5 also interacted with the RING finger ubiquitin E3 ligase KEEP ON GOING (KEG), which is also a negative regulator of ABA signaling. T-DNA insertions in *KEG* resulted in increased levels of ABI5, and KEG E3 ubiquitin ligase activity is required for KEG-mediated regulation of ABI5 abundance [174]. It was suggested that ABA promotes ABI5 accumulation by inducing selfubiquitination and proteasomal degradation of KEG. DWA1 and DWA2, which are components of CUL4-based E3 ubiquitin ligases, interacted with ABI5 *in vivo* and were also suggested to target ABI5 for degradation [174,175]. Furthermore, proteosome-dependent degradation of ABI5 was suggested from analysis of the 26S proteasome subunit, RPN10. The *rpn10* mutant showed hypersensitivity to ABA as well as stabilization of the short-lived ABI5 [176].

The E3 small ubiquitin-related modifier (SUMO) ligase SIZ1 also negatively regulated ABI5 to attenuate ABA-signaling. A [K319R] substitution in ABI5 blocked SIZ1-mediated sumoylation of ABI5 *in vitro* and in *abi5-4* plants *ABI5(K391R)* expression caused greater ABA hypersensitivity than plants expressing wild-type *ABI5* (Figure 2A). The conjugation of SUMO to ABI5 both repressed its activity and prevented its degradation [23,177]. Further studies will reveal whether an interplay exists between ubiquitination and sumoylation in regulating ABI5.

The *Arabidopsis* Mediator subunit MED25, a coactivator of the RNA polymerase, represents a different type of negative regulator of ABI5. MED25 physically associated with ABI5 in promoter regions of ABI5 target genes, which resulted in a negative effect on ABI5-regulated gene expression. Interestingly, MED25 was highly enriched in the *Em6* promoter in the absence of ABA, but in response to ABA treatment MED25 recruitment to the *Em6* promoter decreased [178]. ABI5 also participates in additional interactions, which may play regulatory roles. Thus, ABI5 physically interacted with ABI3, with which it also interacted genetically, using the region of amino acid residues 123–371, and ABI5 and ABI3 were suggested to be part of regulatory complexes of varying composition [138].

### 4.2. DREB2A Interactome and Regulation

Many protein interaction partners have been assigned to the DREB2 interactome (Figure 3B), and some of these are regulators of the DREB2A activity and level. The E3 ubiquitin ligases DREB2A INTERACTING PROTEINS 1 and 2 (DRIP1 and 2) were identified as DREB2A interaction partners and may utilize ID in the *N*-terminal region of DREB2A for these interactions (Figures 2B and 3B). DRIP1 and 2 negatively regulated plant drought stress-responsive gene expression, possibly through targeting of DREB2A for proteasomal degradation [179]. DREB2A also interacted with the regulatory hub protein Radical Induced Cell Death 1 (RCD1) and its closest paralogue SIMILAR TO RCD ONE (SRO1) [180]. RCD1 is degraded upon heat shock and the RCD1-interaction-deficient *dreb2a* splice variant DREB1a.2, accumulated upon heat shock and senescence. It was, therefore, suggested that removal of RCD1 or the loss of the interaction domain in DREB2A is required for proper DREB2A function under abiotic stress conditions [168]. Interestingly, the RCD1-interacting motif in DREB2A is present in an α-helical region [168], which is part of a larger disordered region in DREB2A that may provide flexibility to the regulatory interaction with RCD1 (Figure 2B) [134].

DREB2A interactions with MED25 also involved the C-terminal part of DREB2A (Figures 2B and 3B), which was shown by biophysical analysis to be disordered [144]. Interestingly, binding of DREB2A to both MED25 and the DRE *cis*-acting element resulted in structure induction in DREB2A, and the presence of the DRE *cis*-acting element reduced the affinity of the interaction between DREB2A and MED25. This suggested that transcriptional regulation by DREB2A is facilitated by small but distinct structural changes [144], which may involve structure induction in the ID regions of DREB2A. The additional interaction partners in the DREB2A interactome (Figure 3B) were identified in yeast two-hybrid screenings, and have so far not been characterized further [179,180]. In conclusion, DREB2A regions with a significant degree of ID are implicated in interactions with important regulators of DREB2A level and function. Future research will show whether or how the proteins in the large DREB2A interactome influence the functionality of the stress-associated biochemical hub DREB2A.

### 4.3. ANAC019 Interactome and Regulation

As for other NAC TFs, the interactome of ANAC109 is limited, especially when excluding homo and hetero dimerization, which takes place through the *N*-terminal NAC domain (Figures 2C and 3C) [13,147]. Many interactions can be expected for the large, relatively disordered NAC TADs, but the low number of identified interaction partners may reflect the challenge of identifying interaction partners of self-activating protein regions using yeast two-hydrid screenings. However, the DNA-binding NAC domain is also implicated in interactions with other proteins, as exemplified by the regulatory interactions between ANAC019 or NAC1 and E3 protein ubiquitin ligases [181,182] (Figure 2C). ANAC019 was identified as an interaction partner of the small RING-H2 protein RHA2a [181], which is a functional E3 ubiquitin ligase and a positive regulator of ABA-signaling [183]. Additional RING-H2 proteins also interacted with ANAC019 in yeast two-hybrid assays (Figure 3C). Further studies are needed to analyze if RHA2A or other RING-H2 proteins mediate proteasome-dependent degradation of ANAC019 for example to attenuate ABA signals.

Up-regulation of the *ERD1* gene depended on co-overexpression of ANAC019 and ZFHD1, and this cooperation may depend on the physical interaction between ANAC019 (region 1–186) and ZFHD1 [61] (Figures 2C and 3C). A putative function of the interaction between ANAC019 and *Arabidopsis* CTD phosphate-like1 (CPL1) remains to be determined [184]. Clearly, additional interaction partners can be expected to be identified for ANAC019 and other stress-related NAC TF in the future.

### 4.4. WRKY40 Interactome and Regulation

So far the interactome of WRKY40 is limited to homodimerization and interactions with the closely related WRKY18 and WRKY60 in plant defense responses and the ABA receptor magnesium-protoporphyrin IX chelatase H subunit (CHLH/ABAR) [93,185] (Figure 3D). When the ABA level is high WRKY40 is recruited from the nucleus to the cytosol, where it interacts with CHLH/ABAR localized to the chloroplast/plastid envelope membrane. Thereby CHLH/ABAR mediates de-repression of ABA-responsive genes such as *ABI5* allowing ABA responses [93]. Interestingly, homo dimerization of WRKY40 and interactions with WRKY18 and WRK60 was dependent on a potential leucine zipper region outside the WRKY DBD domain (Figure 2D) [185]. Hetero dimerization of these WRKY TFs have also been suggested to play a functional role in ABA responses. Thus, a WRKY18/WRKY40 hetero complex was suggested to regulate the expression of the *WRKY60* gene, in accordance with the induction kinetics of these three genes [92]. WRKY40, WRKY18, and WRKY60 also had partially redundant negative effects on SA-mediated defense but played a positive role in JA-mediated defense [185]. These WRKY TFs may therefore mediate crosstalk between plant defense and ABA-related signaling.

### 4.5. Zat7 Interactome and Regulation

The EAR motifs of Zat7 (Figure 2E) are implicated in the function and interactions of this repressor. Thus, transgenic plants overexpressing *Zat7* are more tolerant to salt stress than wild type plants, but deletion or mutation of the EAR motifs abolished this increased tolerance. In contrast, the EAR-motif did not affect growth repression reported for *Zat7* overexpressing plants. The interactome of Zat7 is large, but only nine proteins which were regarded potential interactions partners [100] are shown in Figure 3E. Zat7 interacted with stress-responsive and defense-related proteins such as the TF WRKY70 and the miRNA transport protein HASTY through its EAR motifs. *Zat7*, *WRKY70*, and *HASTY* were shown to be co-expressed in plants deficient in the H_2_O_2_-scavenging enzyme ascorbate peroxidase 1 (*apx1*) [186,187]. Since *apx1* plants were also more tolerant to salinity stress, *Zat7*, *WRKY70*, and *HASTY* may be part of the same salt stress signaling pathway [100]. Interestingly, WRKY70 is critical for effective defense against pathogen attack and a key regulator in the antagonistic interaction between SA and JA [188], but also likely to play an important role in abiotic stress responses. In conclusion, the EAR-motifs of the Cys2/His2-type zinc finger repressor Zat7 play a role in abiotic stress responses through interactions with other proteins.

### 4.6. ICE1 and MYB15 Interactomes and Regulation

The cold response in *Arabidopsis* is also attenuated by the proteasome pathway [189]. The variant RING finger protein HIGH EXPRESSION OF OSMOTICALLY RESPONSIVE GENE 1 (HOS1) physically interacted with ICE1 (Figure 3G) and was required for ubiquitination of ICE1. Cold induced degradation of ICE1 was blocked by the *hos1* mutation, and overexpression of *HOS1* in transgenic *Arabidopsis* reduced the expression of *DREB*/*CBF* genes and decreased plant-freezing tolerance [189]. Recently Ser403 of ICE1 was shown to be polyubiquitinated *in vivo* (Figure 2G) and to play a role in both the transactivation activity and the cold induced proteasome-dependent degradation of ICE1 [190].

Like ABI5, ICE1 is a target of SIZ1 [191,192] (Figure 3G). SIZ1-mediated sumoylation of ICE1 at K393 (Figure 2G) facilitated ICE1 activity and stability and, thereby, positively regulated DREB1A/CBF3-dependent cold responses. Furthermore, sumoylated ICE1 repressed the function of both HOS1 and MYB15, which are negative regulators of the *DREB/CBF* genes, from its interactome (Figure 3G). Thus, sumoylation of ICE1 blocked HOS1-mediated poly-ubiquitination of ICE1, as an example of an antagonistic effect of ubiquitination and sumoylation, and downregulated *MYB15* expression [192]. ICE1 remains the only known interaction partner of MYB15 (Figure 3F). Since MYB15 plays a significant role in cold regulation, and possibly also in drought and salt stress regulation, mapping of the MYB15 interactome is of great interest.

Heptahelical protein 1 (HHP1) which is a negative regulator in ABA and osmotic signaling in *Arabidopsis* also interacted with ICE1 and the *hhp1-1* mutant showed hypersensitivity to cold stress with limited watering suggestive of crosstalk between cold and osmotic signaling [193]. SPEECHLESS (SPCH), MUTE, and FAMA of the ICE1 interactome are implicated in stomatal development and possibly related to abiotic stress through their interaction with ICE1 (Figure 3G) [194].

### 4.7. TF Interactomes in Abiotic Plant Stress

Currently data on plant interactomes is accumulating fast from both individual studies and large scale analysis, as apparent for ABI5, DREB2A, ANAC019, and ICE1. E3 ubiquitin ligases are significant components in several of the TF interactomes shown in Figure 3 involving both ABA-dependent (ABI5 and ANAC019) and ABA-independent (DREB2A and ICE1) signaling pathways. This reflects the importance of the ubiquitin-proteasome system (UPS) in removing regulatory proteins, like TFs, when they have become ubiquitin-tagged. This type of regulation allows the cell to respond rapidly to changes such as environmental stress [195]. Thus, interactomes as shown in Figure 3 complement gene regulatory networks as shown in Figure 1 in describing the molecular regulatory mechanisms of abiotic stress responses.

Within the last ten years the ubiquitin-like modifying protein SUMO has been shown to regulate TF level and activity [196]. The significant role of sumoylation of TFs in abiotic stress responses is also demonstrated by the examples highlighted in this review. The E3 SUMO ligase SIZ1 interacted with and regulated the level of both ABI5 implicated in ABA-signaling and osmotic regulation [23], and ICE1, a regulator of the cold responses [192]. Still much remains to be elucidated to improve the understanding of the SUMO-abiotic stress association.

The interactomes in Figure 3 represents additional interesting regulatory interactions. The RCD1-interaction-deficient *dreb2a* splice variant DREB2a.2, which lacks the RCD1-interacting region, accumulated upon heat shock and senescence. This suggested that abolishment of the RCD1-DREB2A interaction is required for DREB2A function, and that RCD1 could mediate DREB2A degradation [168]. This illustrates the importance of both protein level and alternative splicing in TF regulation. The interaction between the ABA-receptor CHLH/ABAR and WRKY40 to abolish repression of WRKY40 target genes such as ABI5 [93], and the EAR-motif-dependent repressor function of Zat7, likely involving PPIs [100], represent different TF regulatory mechanisms.

Figure 2 also shows TF regions involved in interactions with other proteins and positions or regions of post translational modification of the TFs. Sumoylation of ABI5 at position 391 is in accordance with the emerging picture that post translational modifications are often targeted towards regions with ID (Figure 2A) [162]. ICE1 is also both sumoylated and ubiquitinated in disordered regions (Figure 2G). ID is also utilized for protein interactions of ABI5 (Figure 2A), DREB2A (Figure 2B) [134,144], and possibly ICE1 (Figure 2G). The significant degree of disorder in several of the TFs suggests that these TFs are hubs of large interactomes [197]. From analysis of human proteins it is becoming apparent that regions with ID represent suitable target for drugs, such as peptide-inhibitors, which associate with proteins interaction surfaces [198]. Such peptide drugs only interfere with a specific, or a few, of the many potential interactions of large hub TFs and therefore may have well-defined, or more specific, effects compared to those obtained from ectopic TF expression. However, for NAC019, the folded DNA-binding NAC domain is responsible for interactions with both RHA2A and ZFHD1 [61,181]. The capability of the NAC domain to interact with both DNA and other proteins may either be explained by its large size or suggest competition for binding. The relatively small protein Zat7, predicted to be mostly folded also in the protein-interaction EAR-motif region, may also use folded ZFs to expand its interaction potential. In conclusion, although some of the TFs in Figures 2 and 3 can use folded domains for protein interactions, the overall interaction potential of ID regions is clear from the interactomes presented.

## 5. Conclusion

A multitude of examples in section 2 demonstrate that TFs are key regulators of both ABA-dependent and ABA-independent abiotic stress responses. Several examples from the model species *Arabidopsis* demonstrate how ectopic expression of some of the TFs can improve stress tolerance, also without negative effects on growth and development [53,55]. Ectopic expression of specific TFs with the purpose of improving stress tolerance has also been successful in different crop species [10,13,36,53,76]. However, the effect of ectopic expression is not always simple, as shown for Zat10 which may be both a positive and a negative regulator of plant abiotic stress responses [102]. Future efforts in genetic engineering include fine-tuning of the expression of key TFs for stress tolerance in specific temporal and spatial patterns to avoid negative effects in growth and yield. Different types of regulatory proteins such as ubiquitin E3 ligases with specific TF substrates represent alternative future targets of genetic engineering of improved stress tolerance. In this review we also focused on TF structure including ID, which is an emerging scientific topic that deserves attention in future basic and applied plant research. In conclusion, novel strategies may enter plant science in near future to meet agro-economical interests.
